# Gas Chromatography–Mass Spectrometry (GC-MS) in the Plant Metabolomics Toolbox: Sample Preparation and Instrumental Analysis

**DOI:** 10.3390/biom16010016

**Published:** 2025-12-22

**Authors:** Nadezhda Frolova, Anastasia Orlova, Veronika Popova, Tatiana Bilova, Andrej Frolov

**Affiliations:** 1 Laboratory of Analytical Biochemistry and Biotechnology, K.A. Timiryazev Institute of Plant Physiology, Russian Academy of Sciences, 127276 Moscow, Russia; frolovanadja@yandex.ru (N.F.); lanas_95@mail.ru (A.O.); 2 Department of Plant Physiology and Biochemistry, Saint-Petersburg State University, 199034 Saint-Petersburg, Russia; veronika.chantseva@gmail.com

**Keywords:** primary metabolites, GC-MS-based profiling, plant metabolomics, metabolomics platforms

## Abstract

Metabolomics, which is typically referred to as the post-genomic methodology addressing low-molecular-weight metabolites, became a powerful tool in post-genomic research over the last two decades. Indeed, the state-of-the-art metabolomics relies on several well-established complementary platforms—nuclear magnetic resonance (NMR) spectroscopy, liquid and gas chromatography coupled on-line with mass spectrometry (LC- and GC-MS, respectively), and capillary electrophoresis–mass spectrometry (CE-MS). Among them, GC-MS represents one of the oldest and most well-established techniques currently employed in the metabolomics of volatile compounds and non-volatiles—polar low-molecular-weight metabolites, which can be efficiently converted in volatile form by comprehensive derivatization of polar functional groups. Currently, GC-MS is established as the principal analytical method for characterizing primary plant metabolism, although other methods also contribute significantly to determining the complete metabolite profile. Therefore, here, we address the role of GC-MS in plant metabolomics and its potential for the profiling of low-molecular-weight metabolites. Further, we comprehensively review the methods of sample preparation with special emphasis on extraction and derivatization approaches, which are currently employed to improve the method performance and its metabolome coverage.

## 1. Introduction

**Figure 1:**
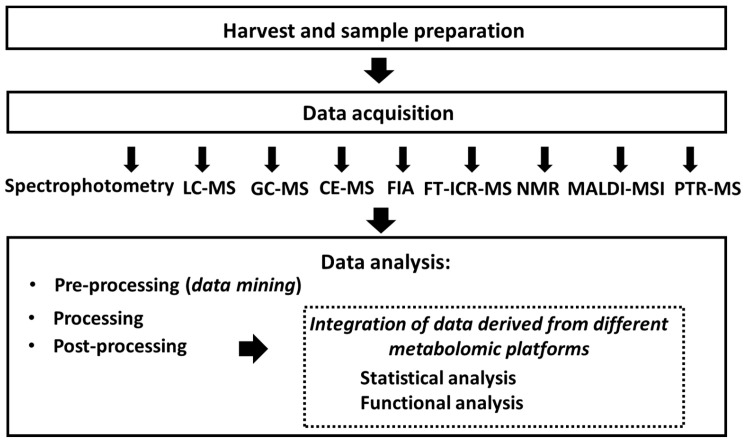
Metabolomics analysis workflow: the generalized schematic overview.

Metabolomics is usually referred to as the post-genomic methodological platform addressing low-molecular-weight (typically below 1 kDa) metabolites present in biological samples [1]. To date, this methodology is well established and is universally recognized as a powerful tool to decipher the plant metabolite network [2,3]. Due to the impressive diversity of plant metabolites (in terms of their molecular weights and sizes, characteristic structure moieties, as well as parameters such as polarity, volatility, solubility, stability, and pK), their analysis appears as rather challenging. Therefore, to cover the whole diversity of natural plant products, several orthogonal analytical strategies need to be employed. To date, this approach is recognized as the only strategy to address this metabolic diversity both at the qualitative and quantitative level [4,5]. Thus, state-of-the-art plant metabolomics relies on a combination of several analytical platforms, which deliver complementary information with subsequent comprehensive data analysis and integration (Figure 1).

Currently, the shortlist of the most widely used metabolomics platforms includes (i) spectrophotometry [6,7], (ii) flow injection analysis (FIA, also often referred to as the direct injection mass spectrometry—direct MS) [8], (iii) gas chromatography coupled on-line with mass spectrometry (GC-MS) [9,10], (iv) liquid chromatography–mass spectrometry (LC-MS) [11,12] and its variant (v) liquid chromatography–high-resolution mass spectrometry (LC-HR-MS/MS), which includes data-dependent acquisition (DDA) and data-independent acquisition (DIA) methods [13], (vi) capillary electrophoresis–mass spectrometry (CE-MS) [14], (vii) Fourier-transform ion cyclotron resonance mass spectrometry (FT-ICR-MS) [13], (viii) nuclear magnetic resonance (NMR) spectroscopy [15,16], (ix) matrix-assisted laser desorption/ionization mass spectrometry imaging (MALDI-MSI) [17], and (x) proton-transfer-reaction mass spectrometry (PTR-MS) [18].

In the post-genomic era, with the development of high-throughput analytical techniques and bioinformatics tools, among the mentioned metabolomics platforms, GC-MS remains a universally available and welcomed method, applicable to a broad range of structurally diverse plant metabolites [19]. Despite GC-MS being a long-established routine analytical method, it has not lost its relevance and is widely used by researchers to provide a robust analysis for a wide range of compounds, allowing their identification and quantification with high accuracy and reliability [10,20]. That is why we are focusing our attention on GC-MS as a methodological platform through a series of three articles. Here, we start by assessing the place of GC-MS in the metabolomics toolbox, evaluating its suitability, applicability, and relevance in comparison with other modern metabolomics methods. Next, we discuss the recent advances in main instrumental techniques applied at the steps of sample extraction, derivatization, introduction, and separation, which significantly enhance the analytical potential of GC-MS in the analysis of diverse plant metabolites. Further critical aspects of the GC-MS analytical workflow with a special emphasis on the selection of the analytical platform for the primary metabolome of plants, data acquisition, the processing of the resulting GC-MS data, as well as its integration with datasets originating from various metabolomics and other omics platforms (genomics, transcriptomics, proteomics), and finally, metabolic pathway elucidation will be discussed in the following two parts of the series.

## 2. Overview of Major Analytical Platforms in Plant Metabolomics

Over the last two decades, metabolomics emerged as a rapidly developing methodology to address the dynamics of plant-cell metabolites (i.e., a group of low-molecular-weight compounds present in plant extracts) in the most comprehensive way [21]. In the following paragraphs, we will provide a brief overview of the main methodological platforms routinely employed in modern metabolomics. Despite the numerous high-throughput methods that enable the establishment of extensive datasets on plant metabolites [17,18,22,23], we will concentrate on a few of them (CE-MS or LC-MS, GC-MS and NMR) in our review, providing a brief assessment of their potential, advantages, and disadvantages. We will then compare the effectiveness of these methods with GC-MS, placing special emphasis on the corresponding separation and detection methods.

### 2.1. Mass Spectrometry (MS)-Based Metabolomics Platforms

MS-based detection methods in combination with various separation techniques provide high sensitivity and flexibility for the metabolomics experiment, giving access to simultaneous detection and quantification of hundreds of metabolites [24].

As small molecules demonstrate essential diversity of physicochemical properties, their annotation relies on several parameters of chromatographic separation and MS-based detection: retention time (t_R_), mass-to-charge ratio (*m*/*z*) of the molecular (adduct) ion, collision cross-section (CCS), tandem mass spectra (MS/MS or multi-staged MS-MS^n^—the *m*/*z* values and relative signal intensities of the fragment ions), and co-elution with authentic standards (i.e., matching of t_R_s, CCSs, and *m*/*z* values of the molecular/fragment ions with those of the standards or references) [25].

Based on the Metabolomics Standards Initiative [26] and according to the criteria proposed by Schymanski and co-authors in 2014 [27], the corresponding annotation confidence levels are currently universally used as follows: (i) metabolites identified by co-elution with authentic standards and confirmation by exact (typically proposed within 3, or even below 1 ppm as the value, giving only one or few elemental composition assignments) *m*/*z* value of the molecular ion (level 1), (ii) presumably annotated by spectral similarity using spectral libraries or by characteristic fragments in MS/MS spectra (level 2), (iii) presumably characterized based on diagnostic ion signals and/or partial spectral similarity with known compounds representing the corresponding chemical class (level 3), (iv) unknown metabolites with characteristic MS profiles and proposed elemental compositions (level 4), and (v) totally unknown species, for which no elemental composition can be predicted (level 5). Here, we briefly address the major methodological platforms of the MS-based metabolomics, paying special attention to their analytical potential in the context of the small-molecule analysis.

#### 2.1.1. Gas Chromatography–Mass Spectrometry (GC-MS)

Martin and James are universally recognized as the pioneers of GC, who reported the first mixture separation by gas distribution chromatography in 1952, based on the results of Martin and Singh [28,29]. After this, GC rapidly became a routine analytical method. A further breakthrough in gas chromatography was the introduction of the flame ionization detector by McWilliam and Dewar in 1957 [30]. At the next step, electron (impact) ionization (EI)-MS coupling was introduced [31]. Already in the early 70s, the concept of “metabolite profiling” was introduced by Devaux et al. [32], who employed it in the analysis of steroids, amino acids, and drug metabolites in human biological fluids. In the following years, GC-MS became the method of choice for metabolomics studies in medical and forensic practice [29]. However, only two decades later, in the late 90s, the concept of metabolite profiling was extended to various plant objects [33,34,35,36,37,38,39]. During this time much attention has been paid to the investigation of secondary metabolites: brassinosteroids (cell culture of *Catharanthus roseus*) [35], phenolic and anethole content in differentiated tissue cultures of *Agrobacterium*-transformed roots of anise [38], essential oils in *Agrobacterium*-transformed roots of *Artemisia absinthium* [39], saponins [40], alkaloids [37], and phytoestrogens [36].

The era of plant metabolomics was opened by the Willmitzer’s group (Max Planck Institute of Molecular Plant Physiology) in 2000–2001 [41,42,43,44,45,46,47,48,49]. Thus, in the work of Fiehn and co-authors [41], metabolite profiling is positioned as a new tool for functional genomics, allowing the determination of “metabolic phenotypes”. In particular, the use of GC-MS allowed the automatic identification of 326 different compounds from *Arabidopsis thaliana* leaf extracts, half of which had their chemical structure determined [41]. Based on elemental composition calculations obtained using gas chromatography–low-resolution quadrupole mass spectrometry, more than 15 new metabolites were detected in the polar fractions of extracts from unpurified *A. thaliana* leaves, e.g., tartronate semialdehyde, citramaleic acid, allothreonine, and glycolamide [42]. The combination of GC-MS with other methods, in particular with the nonaqueous fractionation method, made it possible not only to identify intermediate products of glycolysis, nucleotides, sugars, organic acids, amino acids, and polyols in potato tubers (*Solanum tuberosum* L. cv Desiree) but also to analyze their compartmentalization [49]. All their studies relied on GC coupled on-line with quadrupole MS with EI (GC-EI-Q-MS) at 70 eV, to ensure inter-instrumental/inter-group compatibility and access to reliable library search by spectral similarity [42,46]. The researchers focused mostly on primary metabolism. To make the primary metabolites volatile, appropriate pre-column derivatization methods were employed. Since this time, GC-MS became the method of choice for analysis of plant primary metabolome by the metabolite profiling approach. This approach aims at an exhaustive analysis of low-molecular-weight volatile metabolites and non-volatile metabolites that can be modified into volatile ones by derivatization [50].

**Figure 2:**
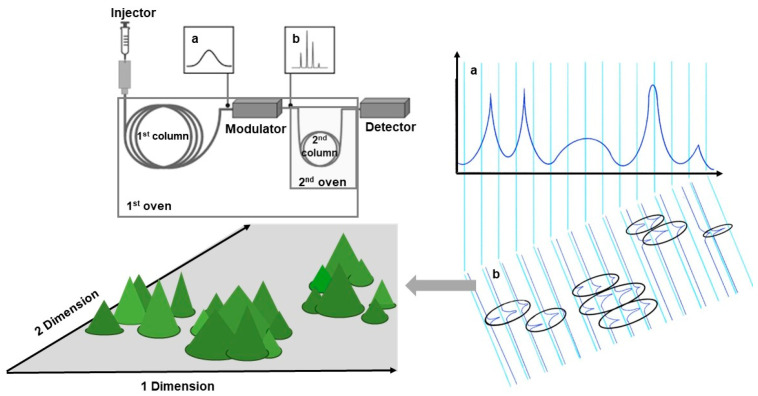
The scheme of a two-dimensional GC-MS instrument and visualization of the resulting chromatogram. Recording of metabolomics data in GC in the first (**a**) and second dimensions (**b**). Circles indicate resolved chromatographic peaks that overlapped during the first GC separation.

Later, it was shown that the metabolite coverage of GC-MS experiments could be significantly enhanced by implementation of multidimensional separations. In the most advanced way, this approach relies on two-dimensional comprehensive gas chromatography (GC×GC): this method combines two orthogonal separation steps that essentially enhance analytical resolution [51]. For the switch between the orthogonal dimensions, a modulator approach is typically employed. This device collects the effluent from the primary column (first dimension) and periodically transfers it to the secondary column (second dimension) as sharp bands, followed by fast two-dimensional separations [52]. GC×GC chromatograms are visualized as contour plots, resolving peaks that would overlap in conventional GC analysis (Figure 2).

In comparison to the single-dimensional approach, this technique offers increased peak capacity without extending analysis time, giving access to reliable qualitative and quantitative analysis. GC×GC coupled with high-resolution (HR) TOF-MS in a comprehensive profiling experiment of four *Eucalyptus* spp. leaf oil extracts resulted in the detection of 400 analytes and identification of 183 of them [53]. However, despite the benefits of multidimensional analysis, which allows the acquisition of robust high-density data, the challenge of data processing in metabolomics investigations persists across different instrumental platforms. In particular, there is a need for accurate annotation and realignment of detected features across multiple samples and the need for spectral databases and appropriate software for processing such multidimensional data [54,55]. Recent studies confirm the effectiveness of GC×GC as an analytical tool in a wide range of applications, particularly for studying potential antimicrobial and biologically active substances in plants and analyzing volatile substances with a focus on practical applications in biomedicine, the food industry, and plant cultivation [56,57,58,59].

Thus, over the last decades, GC-MS remarkably evolved in terms of sample preparation techniques, technical design, performance of instrumentation, and data processing tools. As the state-of-the-art analytical platform, GC-MS requires (i) high-throughput sample preparation methods, including efficient extraction procedures and, if necessary, quantitative and complete chemical modification (derivatization) of target metabolite groups, (ii) avoiding thermal degradation of analytes and their derivatives during analysis, (iii) the optimization of separation conditions to reduce interference with the matrix, (iv) a broad linear dynamic range for simultaneous analysis of major and minor sample constituents, (v) a reliable manual and automatic annotation of metabolites and their reliable quantification, and (vi) efficient bioinformatics and statistical platforms for data processing and post-processing.

To date, most of these requirements are successfully met. Therefore, at the current state of metabolomics, GC-MS is obviously the best-established instrumental platform within this methodology [41]. Indeed, GC-MS ideally combines high precision, accuracy, linearity, and overall reliability of quantification with relatively simple and straightforward protocols for spectral similarity-based identification using in-house, commercial, and open access spectral libraries [60]. This makes GC-MS a “gold standard” in metabolomics, which is quite helpful in answering complex physiological and phytochemical questions [10].

#### 2.1.2. Capillary Electrophoresis–Mass Spectrometry (CE-MS)

To date, capillary electrophoresis coupled on-line with mass spectrometry (CE-MS) ideally combines the advantages of high separation efficiency of CE and rich structural information delivered by MS and represents a powerful analytical technique routinely applied for highly efficient profiling of polar and charged metabolites—amino acids, nucleotides, small organic acids, and sugar phosphates [61,62]. In general, CE-MS features high resolution, sensitivity, quantification accuracy, throughput, and low time and material costs and gives access to both cationic and anionic analytes with only nanoliter sample volumes sufficient for successful analysis [63,64]. Low-flow shell-less interfaces based on a porous tip are promising for increasing sensitivity for metabolomics applications [61,65].

One of the main limitations of CE is the fact that the best performance is usually achieved when analyzing small volumes (<50 nL) of samples, which ultimately leads to a loss in the detection efficiency of low-abundance compounds despite the fact that the advantage of this method (CE-MS) is the relatively low-concentration detection limit (LOD) compared with the concentrations analyzed by (U)HPLC-MS [66].

#### 2.1.3. Liquid Chromatography–Mass Spectrometry (LC-MS)

Liquid chromatography–mass spectrometry (LC-MS) is a hyphenated analytical technique that combines the high separation efficiency of LC (to date, typically high-performance (HP)LC or ultra-high-performance (UHP)LC) with impressive sensitivity, accuracy, and abundance of delivered structural information, characteristic for MS [67]. As most of the plant metabolites are polar (e.g., amino acids, amines, sugars, and carboxylic acids) or semi-polar (nucleotides, polyphenols, sterols, and their derivatives), LC-MS appears to be an appropriate and powerful platform for their efficient and comprehensive analysis [68]. The current development of LC-MS instrumentation strongly depends on the progress of the design of new ionization sources and LC-MS interfaces, which rely on a broad array of soft ionization techniques, typically accomplished under ambient conditions [69,70,71,72,73,74,75].

LC-MS is the method of choice for analysis of plant isolates and comprehensive characterization of their secondary metabolite and semi-polar compounds (phenolics, terpenes, alkaloids) [76,77,78]. Ion-pair (IP)- reversed-phase (RP)-HPLC with alkylamines as ion-pair agents can be efficiently used for the analysis of polar-charged metabolites [79]. Hydrophilic liquid chromatography (HILIC) can be used for the analysis of highly polar and ionic metabolites such as cofactors, non-reducing sugars, sugar alcohols, sugar phosphates, etc. [80]. Additionally, LC–MS/MS plays an important role in the discrimination and assignment of structural isomers [81], for example, for the analysis of monosaccharides, flavonoids, and saponins [8,82,83].

Despite its high accuracy, sensitivity and selectivity, the LC-MS and MS/MS methodology has a limitation in the form of “isobaric interference”, which manifests itself as ion-signal suppression [84]. This effect, which is most pronounced for ESI due to competitive ionization, requires special attention when selecting a chromatographic system and optimizing separation.

### 2.2. Nuclear Magnetic Resonance (NMR) Spectroscopy

NMR spectroscopy is a spectroscopic method ideally suited for structural characterization of organic compounds in plant extracts and other complex mixtures, relying on the interaction of an external magnetic field with the nuclei of elements having a non-zero magnetic moment [85]. NMR spectroscopy per se has already been efficiently employed in a variety of plant studies, for example, to characterize of cell-wall components [86], identify chemical markers associated with the final expression of flower color [87], address the effect of environmental and ontogenetic factors on the metabolism of plant leaves [88], etc.

NMR spectroscopy is developing rapidly, especially with the establishment of LC-NMR coupling [89]. A major trend in the current development of the NMR-based metabolomics platform covers the establishment of compound-specific chemical signatures, quality-control chemical markers, the search for drug precursors in plant extracts [90], and strategies to disclose the chemical diversity of medicinal plants [91,92]. The most representative, comprehensive, and freely available for hosting, distributing, searching, and extracting database, the Natural Product Magnetic Resonance Database (NP-MRD) [91], accounts for almost 41,000 spectral and non-spectral compound-specific entries for individual natural products from more than 7400 different living species.

In comparison to MS, NMR spectroscopy is a time-consuming and relatively low-throughput method [93,94]. However, in combination with further detection methods, for example, MS-coupled techniques, UV/VIS spectroscopy, or X-ray diffraction analysis, it requires minimal sample preparation and, in principle, allows both structure assignment and reliable quantification [95]. The current success in the hardware development behind different plant-metabolomics platforms resulted in the establishment of the integrated LC-MS solid-phase extraction (SPE)-NMR workflow, which proved to be efficient in the analysis of secondary metabolites—flavonoids, glucosinolates, terpenoids, and alkaloids [96].

### 2.3. Efficiency of GC-MS for Compound Separation and Detection in Comparison to Other Metabolomics Platforms

Obviously, these mentioned methods essentially differ in their analytical power, i.e., in the numbers of accessible metabolites (and, hence, the maximum possible metabolome coverage). Among the listed techniques, GC and CE provide the highest separation efficiencies—the corresponding numbers of theoretical plates (*N*) reach 70,000–300,000 for GC on capillary columns (varying depending on their lengths) and up to 200,000 for CE [97]. Moreover, due to the relatively fast kinetics of sorption and desorption, RP-HPLC proved to be a highly efficient tool for the separation of semi-polar secondary metabolites [98,99]. The establishment and broad distribution of UHPLC, also often referred to as “sub-2 µm-particle LC” [100,101], over the last two decades essentially increased the potential of this methodology. Thus, GC, CE, and RP-(U)HPLC are recognized as the methods of choice for coupling with MS, tandem MS (MS/MS or MS^n^), and even FT-ICR-MS and NMR (in the case of liquid chromatography), which are, in turn, the most powerful identification methods delivering abundant structural information [13].

It is important to note that high separation efficiency essentially reduces matrix effects and, thereby, improves both the metabolome coverage and linear dynamic ranges (LDRs) of individual analytes [102]. Therefore, hyphenated techniques such as GC-MS and (U)HPLC-MS currently represent the primary “working horses” of plant metabolomics. On the other hand, due to expensive instrumentation, the necessity for time-consuming optimization procedures, and/or long data-acquisition times, LC-NMR and CE-MS platforms are still less popular in comparison with GC-MS as high-throughput methods for metabolite profiling [103]. Finally, among all hyphenated high-throughput metabolomics techniques, GC-MS represents one of the oldest hybrid methodological platforms, which proved to be outstandingly reliable, reproducible, sensitive, and efficient. Therefore, this methodology has been widely employed for the analysis of plant metabolites of various polarities for ages [60,104]. However, despite such long history, even today, GC-MS still has several important advantageous features in comparison with LC-MS or NMR. Indeed, GC-MS instrumentation is much less expensive and relatively easy in operation. Moreover, due to the typically employed hard electron (impact) ionization, this technique only minimally suffers from matrix effects, i.e., the ionization efficiency of an analyte is much less affected by the presence of co-eluting substances than in most other hyphenated methods.

In general, GC is ideally suited for the separation of volatile organic compounds (VOCs). In this context, GC-MS with a headspace injection is the method of choice for the analysis of gaseous analytes (HS-GC-MS) [105,106]. The sensitivity of this approach can be extended by implementation of solid-phase microextraction (SPME), which gives access to a wide range of VOCs that play an important role in plant–environment interactions [107]. Alternatively, in some cases, GC-MS of VOCs can rely on liquid injection; typically, however, such compounds (in particular, light carbonyls) yield a compromised response with this type of sample introduction [108].

Further, GC-MS was extended to the detection and analysis of non-volatile primary metabolites (amino acids, organic acids, sugars, sugar alcohols, amines, fatty acids, and sterols) after their appropriate derivatization [10,109]. Based on this approach, a complete GC-MS-based metabolite-profiling workflow comprising all steps from plant material collection and sample processing to derivatization procedures, instrument setup, evaluation of resulting chromatograms, and data processing was established.

A summary of the application, advantages, and limitations of the principal analytical methods used in plant metabolomics is provided in Table 1.

Currently, a wide range of various available GC-MS-based analytical techniques allows achievement of the best performance of the method for specific biological matrices, which include volatile and non-volatile metabolites. The main instrumental factors critically affecting the analytical potential of the method are the sample extraction (and, in some cases, derivatization) procedures, introduction method, separation system, and MS conditions. In the following paragraphs, we will discuss principals of extraction and separation in more detail and focus on specific aspects of analyzing volatiles and non-volatile metabolites by GC-MS.

## 3. Sample Preparation

### 3.1. Extraction Methods in GC-MS-Based Metabolomics

Despite the high efficiency of the traditional extraction methods employed in plant metabolomics workflows, in some cases, their performance appears to be insufficient. The major rationales behind employing advanced extraction protocols include (i) increasing the extraction yields, (ii) decreasing extraction times, (iii) improving metabolite stability in the extracts, and (iv) the selective extraction of specific metabolite classes. The necessity of the extraction optimization might also be underlain by the complexity of the sample matrix and by the limitations of the analytical platform [111].

#### 3.1.1. Comparative Characterization of Extraction Methods in GC-MS

Most often, these aims can be achieved by the modification of traditional extraction methods. Thereby, the original extraction setup can be principally changed and/or complemented with different physic-chemical procedures aiming for improvements in phase transfer and analyte recovery (Table 2).

#### 3.1.2. Liquid (Liquid–Liquid) Extraction (LLE)

The most easy and straightforward modification of the overall extraction setup is the switch to multistage extraction schemes, in the easiest case implemented as liquid–liquid procedures. On one hand, this approach allows the use of solvents with contrasting physical properties, for example, to remove non-polar contaminants from polar extracts or vice versa [112]. On the other, solvents with similar properties (e.g., water–alcohol mixtures taken in different solvent ratios) can be used to ensure the quantitative extraction of metabolites from complex plant samples [113,114].

#### 3.1.3. Solid-Phase Extraction (SPE) and Solid-Phase Microextraction (SMPE)

Another sample preparation strategy is solid-phase extraction (SPE), which is based on a more or less specific distribution of analytes between a solid stationary phase (sorbent) and a liquid mobile phase (sample matrix, solution of target components, eluent) through multiple consecutive sorption–desorption cycles [115]. This technique can be considered as a convenient alternative to liquid–liquid procedures and often even appears to be advantageous in multiple aspects. Selecting suitable solid- and liquid-phase parameters for analysis results in shorter extraction times, solvent savings, and higher adsorption efficiency, and therefore, more reliable results. Solid-phase materials are divided into two groups: inorganic materials (silicon dioxide, activated carbon, titanium dioxide, etc.) and organic materials (polymers) presented in the form of disks, cartridges, and syringes, each of which has its own characteristics, advantages, and disadvantages. Thus, it offers impressive plasticity in the selection of specific interactions between analytes and the stationary phase, also giving access to precise tuning of the strength of these interactions with simultaneous removal of potentially interfering matrix contaminants [116]. On one hand, it results in improved purification efficiency; on the other, it provides higher selectivity and precision of quantification accessible in more favorable LDRs in comparison with liquid–liquid extraction [117]. Due to the high specificity of the analyte interaction with the stationary phase, each target compound can be selectively enriched or separated from interfering components [118]. The solid-phase extraction method combined with the GC-MS method is widely used in the pharmaceutical and food industries, and in biological and physiological research for sample purification, reduction in matrix effects, or preliminary fractionation of samples of complex composition [119].

Solid-phase microextraction (SMPE), variation in SPE, combines sampling, extraction, and analyte enrichment within a single solvent-free chemical analysis step [120]. The advantages of SMPE include simplicity, high sensitivity, and specificity. Moreover, due to its generally non-invasive nature, this method is well suited for complex biological applications, which are summarized in the recent work of Jalili et al. [121]. Thereby, due to high efficiency, characteristic for the extraction of volatile components from the tissues or even plant environment, SPME is the method of choice for the analysis of such metabolites [122]. In comparison to liquid–liquid extraction, this method shows superior extraction rates and selectivity.

#### 3.1.4. Supercritical Fluids Extraction (SCFE)

A dramatic increase in extraction efficiency can be achieved by implementation of the supercritical fluid (SCF)-based techniques [123]. The term “supercritical fluid” is attributed to individual substances or their mixtures (typically gases under normal conditions), which are compressed above their critical points [124]. The application of SCFs as environmentally friendly eluents in supercritical fluid chromatography is well established since decades [125]. Similar to gases, such eluents are featured with low viscosities and extremely high diffusion coefficients [126], which ensures favorable sorption/desorption kinetics and underlies the superior efficiency of SCF chromatography [127]. Due to these features, SCFs demonstrate improved solubilizing properties in comparison with regular solvents [126]. These properties make SCFs excellent extractants that are routinely used in the methodology usually referred to as supercritical fluid extraction (SCFE) [128]. SCF is used as a substitute for organic extractants not only in laboratory processes but also in industrial protocols routinely employed in production of health- and wellness-related formulations—in food, pharmaceutical, agricultural, and cosmetics chemistry. However, the application of SCFE in phytochemical analysis is limited by the strongly non-polar character of SCF-based extractants. Fortunately, this limitation can be elegantly overcome by the supplementation of organic co-solvents to classical SCF extractants. Thus, Duba and co-workers employed SCFE for the extraction of grape skin and seeds in a semi-continuous mode using subcritical deionized and degassed water as a solvent [129]. Accurate optimization of the extraction conditions allowed obtaining high yields of polyphenolic compounds for both grape skin and defatted seeds. Recently, SCFE was successfully combined with liquid-phase and solid-phase traps [130,131]. This approach appeared to be promising as a cost-efficient approach for extraction, pre-purification, and fractionation of natural products (primary of polyphenolic nature).

As plant isolates are widely used in food, pharmaceutical, and cosmetics chemistry, minimizing the use of organic solvents for the isolation of natural products has currently become one of the main actual trends in natural product chemistry. For this, implementation of various physical methods to enhance the efficiency of the extraction of natural products from plant material needs to be considered.

#### 3.1.5. Microwave Extraction (MWE)

Microwave extraction is one of such methods, universally employed in natural product chemistry labs worldwide. Microwaves cause dipole rotation in organic molecules that might disrupt hydrogen bonds. The accompanying increase in the ion kinetic energy leads to sample heating, which is underlain by molecular friction associated with the continuous movement of the ions and continuous change of the movement directions. Importantly, the disruption of the hydrogen bonds essentially increases the penetration efficiency of solvents (i.e., their ability to penetrate into the plant matrix) [132]. Multiple studies clearly indicate that this extraction strategy, along with the ultrasonic-associated extraction, is superior in comparison to the traditional methods [133,134].

#### 3.1.6. Ultrasound Extraction (USE)

One of the most straightforward and easy implementations approaches to enhance extraction efficiency is exposure of the plant samples to ultrasound. Ultrasound triggers the formation of cavitation bubbles, which explode upon reaching the point of instability and is accompanied by an increase in temperature and pressure in the reaction mixture. Under these conditions, the plant cell walls can be readily destructed, and the release of metabolites from the cell is enhanced [135].

Comprehensive comparisons of this approach with other extraction methods revealed that its efficiency relied on the same factors as traditional protocols [136,137]. The ultrasonic extraction method was shown to increase the yield of some fatty-acid esters, phenolic compounds, and steroidal compounds during extraction [138,139,140].

#### 3.1.7. Pulsed Electric Field Extraction

Another promising approach to increase extraction yields relies on the pulsed electric field, a non-thermal method widely used in the food industry. This technique relies on a critical electric potential, which is applied to accelerate mass transfer and increase the permeability of cell and tissue membranes [141]. Its applicability to the solvent-free extraction of plant metabolites by tissue pressing was addressed. Thereby, the increase in the extraction yields of target natural products from raw materials was observed [142]. Thus, this method can be considered as a promising alternative approach to extract plant secondary metabolites and, specifically, polyphenolic compounds [143]. Treatment with a pulsed electric field can be efficient when sufficiently high powers within amplitude values of up to 35 kHz are used [144].

It is necessary to consider that the lower amplitudes might be inefficient for the extraction of phenolics from some tissues (for example, grape skin), as in such tissue, polyphenols are localized in deep layers—the hypodermis. According to the literature data, the pre-heating of plant material can contribute to the softening of phenolics-rich tissues and might improve the yields of polyphenols under further exposure to a pulsed electric field [145].

Thus, pulsed ohmic heating, combining the effect of an electric field and elevated (up to 50–90 °C) temperatures, was found to be an effective independent method of extraction, positively affecting the process of release of metabolites from plant cells, as well as an effective method of supplementing the pulsed electric field method [146]. Thus, El Darra and co-workers addressed the potential of this method for the extraction of polyphenols from grape peels. The authors showed that the pulsed ohmic heating preceded by maceration with 30% (*v*/*v*) ethanol increased the yields of grape peel polyphenols by 36% in comparison with the untreated samples extracted under the same conditions [147].

#### 3.1.8. High-Voltage Electrical Discharge Extraction

Another way to achieve enhanced extraction efficiency is high-voltage electrical discharge. This innovative liquid-phase discharge technology developed tremendously over the last decade, finding broad applications primarily in the food industry [148]. This approach is advantageous in comparison with typically used techniques, as it assumes the simultaneous treatment of plant tissues with electrical discharge in parallel to shock waves and turbulence for mechanical destruction, ultraviolet radiation for DNA degradation and inactivation of cell metabolism, and free radical ROS to achieve oxidative damage of cells, which is expected to improve the yields of bioactive natural products [149].

El Kantar et al. found that the use of high-voltage electric discharge for the pretreatment of orange peels increases the yields of polyphenols and sugars during further extraction [150]. In their recent critical review, Li and co-workers [149] summarized the application of this method in phytochemical practice. The authors highlighted that in comparison with the conventionally used aqueous-alcohol extraction, the use of high-voltage electric discharge could increase the yields of multiple classes of polyphenolic compounds (flavan-3-ols, flavanols, stilbenes). Moreover, it can reduce the time required for extraction [151].

### 3.2. Derivatization in GC-MS Analysis of Volatile and Non-Volatile Metabolites

#### 3.2.1. Principal Overview of the Derivatization Protocol

Despite the convenience of GC-MS, only a limited number of the naturally occurring metabolites are volatile and, therefore, compatible with this technique per se. Fortunately, a representative selection of well-established chemical derivatization methods provides an elegant solution to this problem. In general, to increase the hydrophobicity of analytes and to bring them to the volatile state, the hydrophilic functions of polar metabolites can be chemically protected by their nucleophilic functional groups prior to the GC-MS analysis [152]. Thereby, the introduction of protective groups with strong positive inductive effects would dramatically reduce the polarity of corresponding covalent bonds and suppress any polar interactions, including hydrogen bonding [153]. This makes the resulting derivatives highly volatile, which dramatically improves the efficiency of their interaction with the stationary phase, giving access to superior kinetics of sorption/desorption. Thus, the changes in the intra-molecular electron density distribution, which are associated with the derivatization of the analyte molecule, essentially improve its analytical behavior—both in terms of chromatographic separation and detection [154]. Obviously, for non-volatile primary and secondary metabolites, chemical derivatization is mandatory for their reliable detection and quantification by GC-MS.

On the other hand, one needs to keep in mind that derivatization can be applied not only to non-volatiles but also to highly volatile analytes, to make individual metabolites less volatile and to prevent their losses via excessive volatilization during sample preparation. Importantly, in this case, derivatization not only suppresses the phase transfer but also improves the chemical stability of multiple or selected (depending on the strategy used) metabolite classes [152].

#### 3.2.2. Silylation

Among the broad selection of the currently available methods, trimethylsilylation represents the most commonly used derivatization technique [10,155]. This method is well suited for the modification of primary metabolites with the structural moieties containing electron-donating atoms—carboxyl (-COOH), carbonyl (-CO), hydroxyl (-OH), amino (-NH_2_), and thiol (-SH) groups, which appear in the structures of carboxylic acids, amino acids, short- and medium-chain fatty acids, sugars, and sugar alcohols) [152]. The reaction relies on the SN2 mechanism with a nucleophilic attack on the silicon atom. The reaction yields byproducts (*N*-methyltrifluoroacetamide, in case MSTFA is used as the silylation agent, and *N*trimethylsilyltrifluoroacetamide, in case of BSTFA is used), representing good leaving groups. Due to this, the derivatization reaction is fast and quantitative, although the mechanism assumes the formation of a stabilized anionic complex [156].

To date, the most used trimethylsilylation agents are *N*-methyl-*N*-(trimethylsilyl)trifluoroacetamide (MSTFA) and *N*,*O*-bis(trimethylsilyl)trifluoroacetamide (BSTFA). Trimethylchlorosilane (TMCS) and *tert*-butyldimethylchlorosilane (TBDMCS) are frequently supplemented to the silylation reaction as catalysts for the trimethylsilylation reaction, providing the necessary organosilicon radicals for the substitution of acidic protons of polar functional groups [42,157,158]. Additionally, trimethylsilylation typically requires a base such as triethylamine or pyridine to increase the reactivity of the reaction [159].

The resulting trimethylsilyl (TMS) derivatives represent perfect analytes for mass spectrometry with electron (impact) ionization (EI-MS), which is typically employed in GC-MS experiments. Indeed, under EI conditions, they are readily involved in hard fragmentation. Therefore, due to the fact that the TMS moiety represents a good leaving group, the corresponding gas-phase reactions demonstrate high yields of TMS-containing fragments and high intensities of the corresponding fragment signals in the EI-MS spectra. Due to this, the EI-MS fragmentation patterns of TMS derivatives are well defined and well reproducible.

However, despite high analytical power and multiple advantages of the trimethylsylilation approach in combination with GC-MS, it has some intrinsic limitations. Thus, it should be taken into account that acid byproducts generated during the use of derivatization reagents can lead to changes in the composition of the GC capillary column stationary phase [160]. Therefore, it is important to select a column stationary phase suitable for the chosen derivatization. It is known that byproducts produced by silylating agents are less damaging to poly(siloxane)-based stationary phases but should be avoided for PEG-based phases due to the presence of active hydroxyl groups [161]. Secondly, due to high rates of the derivatization reaction and pronounced complexity of biological matrices, combined with fascinating efficiency of the GC separation, in many cases, a compound that was originally present in the sample can appear by several peaks in the total ion chromatogram. This fact makes both the identification and quantification of such analytes challenging [162]. Such behavior can be explained by the incomplete derivatization or/and partial degradation of analytes in the injector, which might result in series TMS derivatives with different numbers of the TMS groups attached [163]. Formation of such artifacts might also accompany the molecular dynamics of analytes and correspond, for example, to different mesomeric variants that were stabilized with derivatization [164].

Despite the significant impact of these factors in the patterns of TMS derivatives in the sample, anomeric transitions characteristic for the sugar molecules remain the primary contributor in their complexity. Indeed, being aldo- and keto-alcohols in nature, in aqueous solutions, monosaccharides readily adopt not only linear (also hydrated) but also several cyclic forms (semi-acetals and/or semi-ketals), which exist in complex anomeric equilibrium. In the trimethylsylilation reaction, each of these maximally six anomers form individual TMS derivatives. Therefore, to avoid this, the sugar-carbonyl function needs to be blocked prior to trimethylsylilation. In analytical practice, it can be achieved by introducing an additional methoxyamination step [165].

**Figure 3:**
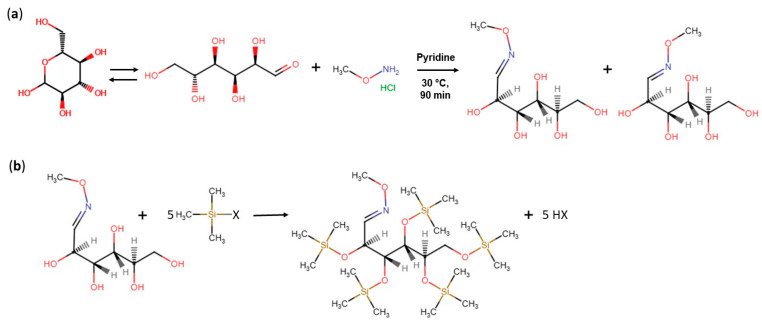
The overview of the derivatization protocol based on the combination of the methoximation (**a**) and trimethylsilylation steps (**b**): the methoximation step with the formation of syn- and anti-stereoisomers (**a**), and trimethylsylilation step with the formation of trimethylsilyl (TMS) derivatives (**b**). X—*N*-methyltrifluoroacetamide or *N*-trimethylsilyltrifluoroacetamide radicals.

At this oximation step, carbonyl groups are converted into the appropriate oximes in the reaction with hydroxylamine or alkylhydroxylamine (*O*-methylhydroxylamine) to stabilize the sugars in the open-ring conformation [166].The oximes exist in the reaction mixture as syn- and anti-stereoisomers, resulting in two chromatographic peaks for each carbohydrate. It essentially facilitates the processing of chromatographic information and makes it more reliable [29] (Figure 3).

Besides trimethylsilylation, multiple other derivatization approaches are available (summarized in Table 2). Interestingly, all these methods share a common mechanism, which relies on the substitution of the mobile proton at the electron-donating atoms with a non-polar protection group that blocks specific reactivity of the original functionality.

#### 3.2.3. Alkylation

One of the most widely used derivatization techniques is alkylation [153]. On one hand, this approach can be used as a stand-alone derivatization method. On the other, it can be employed as the first derivatization step in complex derivatization strategies, where it can be followed with acylation or silylation [167]. It should be noted, that this type of derivatization has important limitations—the alkylation reaction typically requires rather harsh conditions (which might, in some cases, result in decomposition of the target analytes or/and their derivatives), and the alkylation agents are typically very toxic [153].

The most commonly used alkylating agents are represented with dialkyl acetals (e.g., dimethylformamide, DMF) [168]. DMF is used for the esterification of acids to yield their methyl esters. It is the method of choice for carboxylic acids, including amino and fatty acids. It should be noted, however, that this derivatization agent is not applicable for the modification of hydroxyls [153]. As the reaction of acids with dialkyl acetals is rather fast, it is recommended to set the reaction directly prior to the injection. Thenot and Horning recommend dissolving dialkyl acetals in acetonitrile for the derivatization of amino acids and carboxylic acids or in pyridine for the modification of fatty acids directly prior to the reaction at 100 °C [169]. Thenot et al. described another possible modification of the alkylation reaction, which is well suited for the analysis of fatty acids [169]. The authors proposed incubation of the reaction mixtures at 60 °C for 10–15 min directly prior to analysis.

Tetrabutylammonium hydroxide (TBH) can be also used as an alkylation agent for the analysis of carboxylic acids and amines [170]. The reaction products—butyl esters of the analytes—demonstrate distinctly higher (in comparison with the methyl derivatives) retention times. Therefore, this derivatization method is preferred for low-molecular-weight compounds. Importantly, the TBH-based derivatization procedure is fast and does not require high temperatures (i.e., it is quantitative under ambient conditions), therefore, this reagent is well suited for thermally labile metabolites [170].

Boron trifluoride is another efficient and inexpensive alkylation agent, well applicable for organic acids (including fatty acids) [152]. The derivatization reaction is accomplished quantitatively within 5–10 min at 60–90 °C [171]. Application of diazoalkanes as alkylating agents proved to be highly efficient, high-throughput, easy in technical implementation, and attractive in price [152]. This derivatization procedure is well applicable to organic acids (including fatty and amino acids), haloanisoles [172], sulfonylurea derivatives [173], and some herbicides [174]. The use of ferrocenecarboxylic acid chloride as an alkylating agent prior to GC-MS appeared to be efficient in the analysis of alkylphenols—this reaction essentially improved separation efficiency and, therefore, the quality of the metabolite annotation [175]. This derivatization agent helps to minimize the losses of the target compounds through volatilization, adsorption, or contamination by other classes of compounds. In a quick and gentle derivatization procedure, each alkylphenol molecule is tagged with a single iron atom. This increases the accuracy of quantification of individual derivatives in the sample under study (Table 3).

#### 3.2.4. Acylation

Acylation is the third widely used type of derivatization strategy. It relies on the introduction of an acyl group into the structure of a molecule containing mobile hydrogen atoms, i.e., thiols, amines, and alcohols [176]. Carboxylic groups can be also acylated. However, in this case, acylation yields esters, thioethers, and amides [177]. The main advantages of acylation in comparison with other derivatization methods are (i) higher stability of derivatives due to the protection of unstable functional groups, (ii) higher volatility of metabolites, (iii) improved chromatographic separation of metabolites in the mixture, and (iv) well-interpretable fragment signals in the EI-MS spectra.

Other acylating agents are fluorinated anhydrides (trifluoroacetic anhydride, pentafluoropropionic anhydride, heptafluorobutyric anhydride). These compounds show good reactivity with alcohols, amines, and phenols, promoting the formation of stable and highly volatile derivatives [178]. The derivatization reaction proceeds under mild conditions at 50 °C for 15 min in the presence of triethylamine as a catalyst. For metabolites containing hydroxyl and amino groups, fluoroacylimidazoles are recommended as mild derivatizing agents. Among them, trifluoroacetylimidazole, pentafluoropropanylimidazole, and heptafluorobutyrylimidazole are typically used [152]. *N*-methyl-bis(trifluoroacetamide) as a derivatizing agent reacts rapidly with primary and secondary amines and slowly with alcohol and thiols. It is an inert and mild agent recommended for the analysis of sugars, amino acids, and alcohols [179]. The third group of acylating agents includes pentafluorobenzoyl chloride, pentafluoropropanol, and 4-carbethoxyhexafluorobutyryl chloride. These agents are aimed at the gentle derivatization of primary and secondary amines and alcohols, but the derivatization using the latter two reagents proceeds under harsher conditions (75–80 °C) (Table 3).

**Table 3:** Commonly used derivatization methods in the analysis of plant metabolites by GC-MS.

Type of Derivatization	Compound Group	Derivatizing Agent	Reaction Conditions	Result of Derivatization	References
Trimethylsilylation (with or without prior aldo- and keto-group oximation)	Organic acids, amino acids, low-chain fatty acids, sugars sugar alcohols	*O*-methylhydroxylamine *N*-methyl-*N*-(trimethylsilyl)trifluoroacetamide (MSTFA) (typically, in combination with *N*,*O*-bistrifluoroacetamide, BSTFA)	Oximation step: 30 °C, 60 min silylation step: 37 °C, 30 min	Increase in the volatility of the metabolites. Increase in the signal intensity in the total ion current.	[155]
Alkylation	Carboxylic acids, amino acids, fatty acids	Dialkyl acetals	100 °C simultaneously the sample injection 60–90 °C during 10 min	Decrease in the metabolite polarity. Increase in the metabolite stability. Increase in the volatility of the metabolites	[169]
	Carboxylic acids, fatty acids	Tetrabutylammonium hydroxide (TBH)	60–90 °C, 5–10 min		[171,173,174,175]
		Boron trifluoride in methanol or n-butanol	60–90 °C, 5–10 min		
	Fatty acids, amino acids, haloanisoles, sulfonylurea derivative, some herbicides	Diazoalkanes	60–90 °C, 5–10 min		
	Alkylphenols	Ferrocenecarboxylic acid chloride (FCC)	microwave oven heating twice for 30 s with a 60 s break in between		
Acylation	Alcohols, amines, phenols	Fluorinated anhydrides (trifluoroacrtonic anhydride, pentafluoropropionic anhydride, heptafluorobutyric anhydride)	50 °C, 15 min	Increase in the stability of metabolites by protecting unstable functional groups. Increase in the volatility of metabolites. Improvement in the chromatographic separation of metabolites in the mixture. Formation of understandable fragmentation patterns	[180]
	Metabolites containing hydroxyl and amino groups	Fluoroacylimidazoles (trifluoroacetylimidazole, pentafluoropropanilimidazole, heptafluorobutyrylimidazole)	50 °C, 15 min		[179,181,182,183]
	Primary and secondary amines, alcohols, thiols, sugars, amino acids	N-methyl-bis(trifluoroacetamide)	60 °C, 60 min		
	Primary and secondary amines and alcohols	Pentafluorobenzoyl chloride	50 °C, 15 min		
		Pentafluoropropanol	75 °C, 5–10 min		
		4-carbethoxyhexafluorobutyryl chloride	80 °C, 5–20 min		

Thus, derivatization of polar metabolites significantly expands the analytical capabilities of GC-MS in plant metabolomics. The conversion of polar and non-volatile compounds into more volatile and thermally stable derivatives allows for the comprehensive analysis of various metabolites in plant samples, contributing to a better understanding of plant metabolism and the role of specific metabolites in various physiological processes.

## 4. Instrumental Analysis

### 4.1. GC-Based Separation Technique

Generally, GC can be accomplished at the analytical and preparative levels [184,185]. Preparative GC relies on large-diameter columns filled with sorbents with high-loading capacities and aims for the isolation of pure individual volatile compounds in reasonable amounts from complex natural preparations [184]. In contrast, analytical GC most often employs 25–60 m long fused silica capillary columns with typical inner diameters (i.d.) of 0.25–0.32 µm and a thin, solid stationary phase applied on the inner surface of the capillary wall (typically established by liquid-based methods) [186,187]. In this case, the separation relies on the dynamic distribution equilibrium between the gaseous mobile phase and the stationary phase (coating) [188]. The sample can be injected in gaseous phase (head space) or vaporized in a hot injector (liquid injection) [189]. Thereby, the selectivity and efficiency of separation depends on the column parameters (chemical nature and thickness of the stationary phase, column length, and inner diameter) and experimental settings (carrier gas type, gas flow rates, and temperature gradients) [190]. Importantly, any alterations in these parameters might affect analyte separation in a complex and specific way. Thus, the physicochemical properties of column stationary phases play a crucial role in GC analysis. High viscosity, wide liquid range, low volatility, and high thermal stability of the applied stationary phases are essential for optimal performance [191]. The choice of stationary phase directly impacts the maximum allowable operating temperature (MAOT) and lifetime of the GC column. Highly viscous phases are required to maintain coating homogeneity at high temperatures and ensure separation efficiency and column longevity [191]. Modified poly(siloxanes), poly(ethylene glycol) (PEG), and some ionic liquids are commonly used stationary phase chemistries due to their desired properties [191,192].

Additionally, Abraham’s solvation parameter model and the Rohrschneider–McReynolds system can be helpful to ensure the proper selection of the most efficient stationary phase in the application of specific classes of analytes [193,194]. Poly(dimethylsiloxane) phases exhibit low cohesion, favorable cavity formation/dispersion, and high nonspecific retention; while poly(dimethyldiphenylsiloxane) and poly(dimethylcyanopropylphenylsiloxane) phases offer increased dipole-type interactions and hydrogen-bond basicity. PEG-based phases, although hydrogen-bond basic and dipolar/polarizable, generally have lower MAOT values [161]. The polarity number scale normalizes column polarity with respect to a reference column, aiding analysts in column comparisons [195].

Additionally, the desired efficiency and selectivity (and hence, peak capacity) of capillary columns can be adjusted by varying the column length (in the available range of 5–150 m). For less complex samples, relatively low peak capacities would be sufficient, i.e., shorter columns/run times can be employed, and higher throughput can be achieved. The samples of higher complexity (e.g., plant extracts) require better separation efficiencies and peak capacities, which are typically achieved by employing longer columns. However, it needs to be considered that increasing the column length would result in longer analysis times and, hence, higher overall analysis costs. The to-date collected analytical experience clearly indicates that the absolute majority of available biological matrices can be successfully separated on columns with lengths not exceeding 30 m [196]. The i.d. of the column directly affects its capacity (i.e., sample load), which increases with higher diameters. Thus, the capacities of 30 m long columns with 0.53 mm i.d. (stationary phase thickness of 0.25 μm) are in the range of 1000–2000 ng, whereas the possible loads are only 35–70 ng for columns with 0.2 mm i.d. and when the same stationary phases are used. On the other hand, at lower i.d. values deliver higher efficiency and, hence, resolution, whereas the increase in this parameter leads to compromised resolutions. Columns with larger i.d. require less carrier gas inlet pressure to achieve the same linear flow rate. To date, dimethylpolysiloxane columns with i.d. of 0.25 mm are recognized as the standard for the analysis of plant metabolites. This selection allows achieving optimal resolution in reasonable analysis times [197]. The importance of other parameters for the efficiency of GC-based separation is discussed in detail in many works [185,198].

In the following chapters, we address the main directions and mainstreams of the GC-MS development in more detail with special emphasis on its application to the analysis of plant metabolites.

### 4.2. GC-MS in Metabolomics of Volatiles

Gas chromatography–mass spectrometry (GC-MS) is one of the most well-established efficient and robust instrumental platforms for the analysis of volatile compounds—short-chain esters, fatty acids, alcohols, aldehydes, and terpenes [199,200]. Accordingly, GC-MS is recognized as the undisputed method of choice for the analysis of volatile metabolites, many of which are the products of the mevalonate and non-mevalonate pathways of terpenoid biosynthesis, products of phenylpropanoid [201] and lipoxygenase pathways. The latter, such as C6 and C9 aldehydes [202], and the alcohol products of their reduction [203,204] constitute an abundant and diverse class of regulatory, adaptive, and protective metabolites [205,206].

In the plant physiology and ecology context, volatiles play several important roles. First, they are known to protect plants from herbivores by deterring herbivores themselves or attracting their enemies [207]. Also, these compounds can act as attractants for insect pollinators [208]. Besides this, volatile metabolites underlie the mechanisms behind plant communication [209]. It was found that the composition of volatile metabolite mixtures secreted by plants dynamically changed in response to external stimuli [210]. Thereby, each stimulus triggers specific shifts in the patterns of volatile metabolites, yielding characteristic “metabolic fingerprints” of corresponding impacts [211,212]. Additionally, the natural metabolism of volatiles associated with ripening, germination, aging, and microbial contamination during long-term storage should be taken into account.

Knowledge about the metabolic shifts accompanying these processes can give access to the biomarkers for selecting and controlling industrial-storage conditions [213]. Due to the high sensitivity and robustness of both GC and electron ionization (EI)-MS, GC-MS might be a convenient and reproducible platform for routine analyses of such markers [214]. On the other hand, natural volatiles often appear to be commercially important and are widely used in different aspects in human households and everyday life [215]. For example, volatiles comprise the impressing majority of molecules used by the perfume and food industries [216]. Moreover, the high potential of volatiles in the pharmaceutical industry is well confirmed to date [217]. For example, the presence of radical scavenging [218], antimicrobial [219], antimutagenic [220], anticholinesterase [221], anti-inflammatory [222], and anti-tumor [223] activities was clearly demonstrated for essential oils. In addition, essential oils were shown to improve cognitive function and appeared to contribute to stress tolerance [224].

Since GC-MS is one of the most straightforward and commonly used methods for the analysis of volatile metabolites, it is important to highlight some standardized and new trends in the instrumental design developed specifically for volatile compound analysis.

Injection is the first step strongly impacting the overall success of the analysis. Generally, GC injection of volatile compounds can be accomplished in gas or liquid phase [225]. Gas phase (headspace) injection is the most suitable method for analysis of volatile substances, in particular, volatile isoprenoids, carotenoid-derived aroma compounds, volatile phenylpropanoids/benzenoids, glycosidically bound VOC, and alkanes [226,227,228,229]. Headspace technology is a non-destructive method for the introduction of volatile analytes in the GC system. In comparison with solvent-based extraction with subsequent liquid injection, the profiles of volatile metabolites acquired in experiments with gas-phase sample introduction techniques appear to be much closer to the naturally occurring patterns of such compounds. Thus, this strategy of volatile analysis can be considered as the most suitable for environmentally relevant applications [225,226].

The application of liquid split/splitless injection (SSI, 1–2 µL) can be considered as an alternative method for volatile analysis [230]. In this case, the extraction of volatile metabolites from plant tissues relies on non-polar organic solvents, such as hexane, dichloromethane, chloroform, or when the hydro-distillation method (a classic method for the extraction of aromatic compounds from plant material with water and steam, used as an alternative to extraction with organic solvents) is applied. Additionally, liquid injection can rely on the accumulation of substantial amounts of dissolved analyte (for example, aliphatic compounds, aromatics, mono- and sesquiterpenoids, and nitrogen-bearing compounds) in a trapping device with subsequent programmed thermo-desorption [231]. Although the operation temperature of the SSI injector is typically set lower for volatile compounds (50–250 °C instead of 220–260 °C commonly used), it is critical to consider the possibility of sample degradation. Thereby, a split system allows injecting larger volumes of highly concentrated sample without overloading (which is typically accompanied with peak broadening and selectivity loss) and contamination of the column [232,233].

Another important aspect is the selection of an appropriate capillary column. Volatiles (for example, essential oils of *Tanacetum vulgare* L.) are usually separated on capillary columns with dimethylpolysiloxanes (e.g., DB-1, DB-5, CPSil 5) or polyethylene glycol polymers (for example, sesquiterpenes from mechanically damaged leaves of *Centaureinae* species) (Carbowax^®^ 20M, DB-Wax and HP-20M) as stationary phases depending on the polarity of analyzing volatiles [225,234,235,236].

Despite essential progress in the development of the methodology for volatile analysis, this technique is still challenging in reliable operation and suffers from some intrinsic limitations. First, some classes of volatile compounds, like terpenes, terpenoids, some phenol derivatives, and esters, demonstrate pronounced chemical instability, which makes the analysis of such metabolites by GC-MS practically impossible [237]. Secondly, due to their high volatility, the target compounds can be easily lost from the samples during the sample preparation step [238,239]. For this reason, additional chemical derivatization can be employed to increase the stability of such metabolites. This aspect has been discussed in more detail in Section 3.2.

### 4.3. GC-MS in Metabolomics of Non-Volatiles

Although solubility in the carrier gas is a pre-requisite for successful GC analysis, GC-MS proved to be a powerful platform for the analysis of non-volatile low-molecular-weight metabolites. Indeed, although phenolics [240], di- and triterpenes [241,242], alkaloids [243,244], long-chain fatty acids, and oxylipins [245] can be efficiently separated by RP-(U)HPLC, water-soluble, highly polar, non-volatile natural products can be reliably analyzed by GC-MS. Most of these molecules fall into the group of primary compounds, i.e., intermediates of the primary (household) metabolism—such as amino acids, mono- and disaccharides and their derivatives, intermediates of the Krebs and Calvin cycles, the pentosophosphate pathway, shikimate, mevalonate- and non-mevalonate pathways of isoprenoid biosynthesis, fatty acids and their derivatives, etc. GC-MS-based metabolite profiling allows addressing the dynamics of each individual primary metabolite in the context of ontogenesis, responses to stress, or vital stimuli [246].

Based on this information, the involvement of specific metabolic pathways in certain physiological responses can be assessed and comprehensively characterized. These results might give access to the mechanisms behind the corresponding physiological phenomena. Therefore, GC-MS-based primary metabolomics represents a useful and promising tool to address a broad range of plant physiology problems including characterization of plant responses to stress [10,247]. For example, this methodology was successfully employed in the analysis of plant responses to drought—a major factor reducing crop productivity worldwide [248]. Dehydration is known to trigger metabolic adjustment—the accumulation of primary metabolites (predominantly sugars and amino acids) [249] in the background of oxidative/glycoxidative stress [250] and dramatic shifts in plant-carbonyl metabolism [251]. Cold and heat stress are also known to induce pronounced metabolic shifts [109,252]. Both stresses induced an up-regulation of a pool of amino acids derived from pyruvate and oxaloacetate and polyamine precursors [253].

These examples clearly indicate that such metabolomics surveys (especially in integration with other analytical techniques) provide new insights into the mechanisms of plant adaptation to stress at the level of the metabolome. Therefore, the role of individual metabolites as stress protectors can be addressed in a very efficient way. Interactions between plants and different microorganisms is another aspect, which was comprehensively addressed by GC-MS-based metabolomics during the recent decade, providing valuable insights into the impact of symbiotic relationships on the crop-plant productivity [254,255].

It needs to be mentioned that besides its importance in physiological studies, GC-MS of primary polar metabolites can efficiently complement the profiling of secondary plant metabolites, i.e., natural products, potentially promising as biologically active compounds [256,257]. Indeed, the parallel profiling of primary and secondary metabolites with GC-MS and LC-MS, respectively, can be accompanied by in-depth phytochemical analysis with the isolation of major metabolites from plant extracts and assignment of their structures by multistage tandem mass spectrometry (MS^n^) and NMR spectroscopy [258]. The acquired information can complement data on the biochemical diversity of metabolites (which are often not well-systematized) [259]. Not less importantly, this approach might help in answering a number of applied questions related to the standardization of plant material for the food industry and medical practice [260]. In the biotechnology context, such combined metabolomics data allow understanding the influence of growth conditions and environmental factors on biosynthetic pathways and the accumulation of individual groups of metabolites [9]. Finally, in the pharmaceutical context, metabolomics data are useful in the search for natural products, promising as new highly efficient and safe drug candidates [261].

Despite the high potential of GC-MS-based profiling of primary metabolites in answering complex biological questions, this analytical platform has some intrinsic limitations. The most important of them is the non-volatile nature of most of the primary metabolites [262,263,264], i.e., the majority of primary metabolites, such as carbohydrates, amino acids, and organic acids, are highly polar (or even charged) and cannot be efficiently retarded on capillary GC columns. This fact makes it practically impossible to analyze them with GC-MS directly without any modification of their structure prior to analysis. Thus, the probing of plant primary metabolomes with GC-MS assumes the analysis of derivatized compounds. Although chemical derivatization increases volatility and the thermal stability of the analytes, it obviously makes the processing of mass spectra and structure elucidation more challenging.

## 5. Conclusion Remarks

In summary, it should be noted that due to the progressive development of efficient sample harvest, extraction, derivatization methods, and advances in chromatographic/mass-spectrometric technologies, GC-MS data gives access to valuable information on basic groups of volatile non-polar metabolites and non-volatile polar ones. The latter are well known as the main contributors to the primary metabolome. After the appropriate derivatization, these compounds can be efficiently retained and separated on capillary GC-MS columns as corresponding TMS(MEOX) derivatives. However, by using standard protocols adapted for a wide range of biochemical structures, individual metabolites or their groups may be lost due to generalized extraction and derivatization conditions, which negatively affect compound recovery rates. Furthermore, using a well-known low-resolution GC-MS system (GC-EI-Q-MS) to analyze metabolites (e.g., phytohormones) present in plants at low concentrations can be challenging due to sensitivity issues (signals lost in background noise). This may lead to a decrease in the reproducibility of mass spectra and the identification significance of the results obtained. Therefore, global metabolite profiling, especially in complex mixtures, using GC-MS requires strong control of sample processing and analysis conditions to minimize data scatter and ensure quantitative assessment. Moreover, an essential part of primary metabolomes (mostly represented by nucleotides, sugar phosphates, vitamins, hormones, and polyphenols) is still not covered by GC-MS methodology mostly because of their compromised thermal stability and/or high molecular weights. High-resolution GC-MS systems (e.g., TOFMS and Orbitrap instruments) significantly affect the quality of the mass spectrum, increasing accuracy, sensitivity, selectivity, and analytical flexibility of the GC-MS method. In addition, the modern development of portable chromatography systems and the application of algorithms based on artificial intelligence and machine learning for data analysis may contribute to reducing labor costs, increasing throughput and improving accuracy. To ensure the comprehensive analysis of metabolic networks, the acquired GC-MS datasets need to be integrated with the results obtained with other metabolomics instrumental platforms. In addition, such integrated metabolomics datasets can be further combined with data acquired with other omics techniques, such as transcriptomics or proteomics. This will provide much deeper and more complete insights into the understanding of plant metabolism and its regulation at a systems-level.

## Figures and Tables

**Table 1:** Principal analytical platforms used in plant metabolomics.

Analytical Method	Applications	Advantages	Limitations
NMR	Structural identification of metabolites in samples without prior separation into individual components [86,87,88,90,91,92,110]. Analysis of secondary metabolites—flavonoids, glucosinolates, terpenoids, and alkaloids [47].	Requires minimal sample preparation.	Time-consuming and low-throughput method. Signal overlap. The complexity of identifying structures in mixtures.
CE-MS	Highly efficient profiling of polar and charged metabolites—amino acids, nucleotides, small organic acids, and sugar phosphates [56,57].	High resolution, sensitivity, quantification accuracy, throughput, low time and material costs.	Low detection efficiency for low-concentration compounds.
LS-MS	Polar thermolabile metabolites (amino acids, amines, sugars, and carboxylic acids), secondary metabolites and semi-polar (nucleotides, polyphenols, sterols, and their derivatives) [67,68,76,77].	High separation efficiency and impressive sensitivity, accuracy, and abundance.	Matrix effect and limitations on compound identification.
GC-MS	Polar thermostable metabolites; volatile compounds—short-chain esters, fatty acids, alcohols, aldehydes, and terpenes [109].	High selectivity and sensitivity. Reproducible retention time. Rapid identification of compounds using existing spectral libraries.	High complexity, and duration of sample preparation. Loss of water-soluble volatile compounds. The effect of temperature on samples.

**Table 2:** Comparative characterization of extraction methods employed in GC-MS workflows.

Type of Extraction	Mechanism of Extraction	Group of Compounds
Liquid (liquid–liquid) extraction (LLE)	Direct mass transfer from the plant matrix (or crude extract) to the used solvent	Suitable for a wide range of primary and secondary metabolites (phenols, terpenoids, and esters)
Solid-phase extraction (SPE)	Sorption of target components on sorbents of different selectivity with subsequent desorption	Primary and secondary organic compounds and volatile metabolites (essential oils, terpenes), metal–organic compounds, hormones
Supercritical fluids extraction (SCF)	Based on the high solubility of various compressed gases, which can be comparable to the solubility of liquid organic solvents	Phenolic compounds, terpenes and terpenoids, alkaloids, oils, polyunsaturated fatty acids, vitamins, carotenoids, biogenic amines, polyacetylenes
Microwave extraction (MWE)	Creation of dipole rotation in organic molecules and heating, which leads to the breaking of hydrogen bonds	Alkaloids, flavonoids, phenols, steroids, anthraquinones, carotenoids, triterpenoid saponins, oligosaccharides, essential oils, lipids, ascorbic acid, glucosides
Ultrasound extraction (USE)	Generation of cavitation bubbles by ultrasound, which explode when the instability point is reached, creating high temperature and pressure in the reaction mixture, which has a destructive effect on plant cell walls and enhances the release of metabolites from the cell	Phenolics, flavonoids, thymols, saponins, lipids, pigments
Pulsed electric field extraction	Acceleration of mass transfer, increase the permeability of cell and tissue membranes due to the application of critical electric potential	Polyphenols, lipids, pigments, anthocyanins, oligosaccharides, tocopherols, terpenes
High-voltage electrical discharge extraction	The action of electric discharge on the treated material, creating shock waves and turbulence, resulting in mechanical tissue damage, ultraviolet radiation damaging DNA and inactivating cells, and free radicals damaging cells through oxidation, which increases the yield of biologically active substances	Polyphenols, volatile metabolites, stilbenes, oils, reducing sugars, oligosaccharides, terpenes

## Data Availability

No new data were created or analyzed in this study. Data sharing is not applicable to this article.

## Abbreviations

APCI	Atmospheric pressure chemical ionization
APGD	Atmospheric glow discharge ionization
API	Atmospheric pressure ionization
APPI	Atmospheric pressure photoionization
BSTFA	Bis(trimethylsilyl)trifluoroacetamide
CE	Capillary electrophoresis
CE-MS	Capillary electrophoresis–mass spectrometry
DESI	Desorption electrospray ionization
DIA	Data independent acquisition
DMED	2-dimethylaminoethylamine
ESI	Electrospray
FCC	Ferrocenecarboxylic acid chloride
FIA	Flow injection analysis
GC-EI-Q-MS	GC–quadrupole mass spectrometry with EI ionization
GC-FID	Gas chromatography–flame-ionization detector
GC-MS	Gas chromatography–mass spectrometry
HILIC	Hydrophilic interaction chromatography
HS-GC-MS	Headspace injection gas chromatography–mass spectrometry
IC-MS/MS	Ion chromatography–tandem mass spectrometry
IP-RP-HPLC-MS/MS	Ion-paired reversed-phase high-performance liquid chromatography coupled with tandem mass spectrometry
LC-HR-MS	Liquid chromatography–high-resolution mass spectrometry
LC-NMR	Liquid chromatography coupled with nuclear magnetic resonance
LC-MS-SPE-NMR	Liquid chromatography–quadrupole mass spectrometry coupled with solid-phase extraction nuclear magnetic resonance
LDR	Linear dynamic range
MALDI-MSI	Matrix-assisted laser desorption/ionization mass spectrometry imaging
MEOX	Methoximation
MS	Mass spectrometry
MS/MS (MS^n^)	Tandem mass spectrometry
MSTFA	N-methyltrifluoroacetamide
MWE	Microwave extraction
NMR	Nuclear magnetic resonance
NP-MRD	Natural Product Magnetic Resonance Database
PTR-MS	Proton transfer reaction–mass spectrometry
RP-LC-MS	Reversed-phase liquid chromatography–mass spectrometry
RP-HPLC	Reversed-phase high-performance liquid chromatography
SCF	Supercritical fluids extraction
SMPE	Solid-phase microextraction
TBDMCS	tert-butyldimethylchlorosilane
TBH	Tetrabutylammonium hydroxide
TMCS	Trimethylchlorosilane
TMS	Trimethylsilyl
QqQ-MS	Triple quadrupole mass spectrometers
QqTOF-MS	Quadrupole time-of-flight mass spectrometry
UHPLC	Ultra-high-performance liquid chromatography
UV-VIS	Ultraviolet–visible spectroscopy
USE	Ultrasound extraction
VOCs	Volatile organic compounds
